# Foot rotation and the risk of falls in older women: A cross-sectional study

**DOI:** 10.1371/journal.pone.0239065

**Published:** 2020-09-14

**Authors:** Mario Kasović, Lovro Štefan, Martin Zvonar

**Affiliations:** 1 Department of General and Applied Kinesiology, Faculty of Kinesiology, University of Zagreb, Zagreb, Croatia; 2 Department of Sports Motorics and Methodology in Kinanthropology, Faculty of Sport Studies, Masaryk University, Brno, Czech Republic; 3 Department of Physical Activity and Fitness, Faculty of Science, Masaryk University, Brno, Czech Republic; University of Tasmania, AUSTRALIA

## Abstract

Although previous evidence has shown that deviated foot structure and function are associated with falls, little is known of the association between foot rotations and falls in apparently healthy older adults. Therefore, the main purpose of the study was to determine the associations between foot rotation and falls. In this cross-sectional study, we recruited 120 older women (mean±SD; age 71.01±6.77 years; height 158.92±21.41 cm; weight 70.29±12.97 kg; body-mass index 26.79±4.42 kg/m^2^). Foot rotations were assessed by using pressure platform (Zebris manufacturer, Munich, Germany), while the risk of falls was assessed by using Downtown Fall Risk Index questionnaire. Correlations and multiple regression models were applied to calculate the associations. In unadjusted model, higher foot rotation was associated with higher risk of falls (*β* = 0.14, *p*<0.001 for both feet). In a model adjusted for age, body-mass index, foot pain and fitness index, higher foot rotation remained associated with higher risk of falls (*β* = 0.10, *p*<0.001 for both feet). Our study shows that older adults with higher foot rotation are at higher risk of falls. Special interventions aiming to correct for deviated foot function in older women are warranted.

## Introduction

Falls are considered one of the most frequent causes of injuries among the older population [1]. Around 30% of people aged ≥65 years fall annually, while for the people >70 years of age, this percentage increases up to 40% [2]. Falls can cause multiple health-related consequences, including reduced quality of life [3], loss of confidence [4], functional dependence, fractures and high level of morbidity [5].

The incidence of falls has always been associated with gait abnormalities and disturbances [6]. During standing or walking, the foot represents the only contact with the ground and factors that can influence foot structure and function may increase the risk of falls [7]. In a community-based prospective study, Mickle et al. [7] showed that people who fell during the period of 12 months generated a significantly higher peak pressure and pressure-time integral under the foot, compared to non-fallers. Falls can also cause different spatiotemporal disturbances of the foot, including slower gait velocity, shorter step and stride length, and longer contact time [8].

Among several foot factors associated with falls, little evidence has been provided regarding foot rotation and the risk of falls [9–13]. A study by Awale et al. [9] showed that foot pain and foot posture were significantly associated with the risk of falls, where participants with a planus foot posture had increased odds of recurrent falls compared to those with the normal foot posture. Another study has shown, that compared to older adults who did not fall, fallers exhibited decreased ankle flexibility, more severe hallux valgus deformity and decreased toe plantar flexor strength [10]. Reduced ankle flexibility in people who fell has also been reported previously [11, 12]. Finally, Mecagni et al. [13] found significant associations between ankle range of motion and Performance-Oriented Mobility Assessment and functional reach score. According to aforementioned, only a handful of studies have examined the associations between foot rotation and the risk of falls in older adults. Since higher foot rotation is associated with reduced balance and ability to perform functional tasks [10], it is necessary to explore and confirm whether higher foot rotation may be a significant predictor of the risk of falls. In that way, community-based special interventions aiming to correct for deviated foot function are warranted.

Therefore, the main purpose of the study was to determine the associations between foot rotation for both feet and the risk of falls in community-dwelling older adults.

## Materials and methods

### Study participants

In this cross-sectional study, we recruited older adults aged ≥60 years. The study protocol and sample size collection are based on our previous work [14]. In brief, we recruited 210 free-living participants aged ≥60 years from five neighborhoods in the city of Zagreb. The inclusion criteria for participating in the study were: (i) being ≥60 years old, (ii) living independently in the community, (iii) passing the Short Portable Mental Status Questionnaire, (iv) being able to ambulate for at least 10 m with or without an aid, (v) being free from neurological diseases, and (vi) could arrange their own transport to a testing venue in their community. After the initial screening, 120 participants fulfilled the criteria for participation in the study. All participants had given a written informed consent before entered the study. The procedures performed in this study were anonymous and according to Declaration of Helsinki, also approved by the Ethical Committee of the Faculty of Kinesiology, University of Zagreb, Croatia (Ethical code number: 2019).

### The risk of falls

To assess the risk of falls, we used Downtown Fall Risk Index [15], a reliable and valid instrument to measure five modules as follows: (1) previous falls, (2) medication, (3) sensory deficits, (4) mental state and (5) gait. This results in 11 different risk factors, which are then summarized and give the score between 0 and 11. Higher score indicates higher risk of falls [15].

### Foot rotation

To assess the level of peak plantar pressure, we used a Zebris plantar pressure platform (FDM; GmbH, Munich, Germany; number of sensors: 11.264; sampling rate: 100 Hz; sensor area: 149 cm × 54.2 cm). The testing protocol was followed by previous published work [14]. In brief, the participants were told to walk naturally across the platform. Shoes and socks were removed before the testing. When walking, they were required to look straight forward, trying not to target the pressure platform. When they completed the first trial (reaching the end of the walkway), they needed to turn 180° around and continue to walk again over the platform. Finally, when they reached the end of the second walkway (second trial). The protocol was then repeated once again. At the end, they walked four times across the pressure platform and the software generated the data regarding foot rotation for both feet. Of note, the reliability between the trials was high (Cronbach’s α = 0.93).

### Covariates

Age was self-reported. Height and weight were objectively measured (using a Seca portable stadiometer and scale). Body-mass index was calculated as follows: weight (in kg)/height (m^2^) and expressed in kg/m^2^. Presence of foot pain was determined by using the same question, as in previous studies: ‘On most days do you have pain, aching, or stiffness in either of your feet?’ [9]. Responses were categorized as: ‘Yes, pain in one foot’, ‘Yes, pain in both feet’, ‘Yes, but unable to detect the side’ and ‘No, no pain in either foot’. Finally, responses ‘Yes, pain in one foot’, ‘Yes, pain in both feet’ and ‘Yes, but unable to detect the side’ collapsed in the ‘foot pain’ category vs. ‘no foot pain’ category. The Senior Fitness Test was used to assess the level of physical fitness [16]. The measurement protocols and the reliability and validity properties are described elsewhere [16]. In brief, The Senior Fitness Test is composed of seven tests as follows: (1) height and weight, (2) chair stand in 30 s, (3) arm curl in 30 s, (4) 2-min step test, (5) chair sit-and-reach test, (6) back scratch test, and (7) 8-foot up-and-go test. In addition, body-mass index has been proposed as an indicator of general adiposity. However, previous studies have shown that waist circumference may be more reliable and valid measure for assessing adiposity in older adults [17]. Therefore, we measured waist circumference between the last rib and umbilicus.

### Data analysis

Basic descriptive statistics are presented as mean ± SD or median (25^th^–75^th^ percentile range) for normally and non-normally distributed variables. Lastly, the presence of foot pain is presented as the percentage. We calculated *z*-scores for each physical fitness test [14]. To get an overall fitness index, we summed all *z*-scores. The *z*-score was calculated by subtracting participant’s score in the test with the overall mean score of the whole sample and dividing it by the standard deviation. The *z*-score tells us how many standard deviations an individual score is from the mean. Spearman coefficients of correlation and multiple regression models were used to calculate the associations between foot rotation and the risk of falls. Since there were significant differences between the feet in foot rotation variable, we presented the associations separately for the left and the right foot. In an unadjusted model (model 1), we calculated separate associations between foot rotation and potential covariates with the risk of falls. Model 2 was adjusted for age, body-mass index, foot pain and fitness index. The results are presented as *β* coefficients with 95% confidence interval (95% CI). All analyses were performed in Statistical Packages for Social Sciences version 23. (SPSS Inc., Chicago, IL, USA) with statistical significance of *p*≤0.05.

## Results

Basic descriptive statistics of the study participants are presented in Table 1. Some variables regarding Table 1 have been published previously [14]. As expected, body-mass index indicated that the participants were categorized as ‘overweight’. Wilcoxon signed-rank test showed significant differences in foot rotation between the feet (*Z* = -2.789; *p* = 0.005), where the right foot rotated more outwardly, compared to the left foot (Table 1). More than half of the participants reported foot pain.

**Table 1:** Basic descriptive statistics of the study participants (*N* = 120).

Study variables	mean ± SD
**Age (years)**	71.01±6.77
**Height (cm)**	158.92±21.41
**Weight (kg)**	70.29**±**12.97
**Body-mass index (kg/m**^**2**^**)**	26.79±4.42
**Downtown Fall Risk Index (scale)** *	2.00 (1.00–3.00)
**Foot rotation-left foot (º)** *	8.20 (4.50–14.25)
**Foot rotation-right foot (º)** *	9.95 (6.10–15.25)
**Foot pain (% of ‘Yes’ response)** **	54.20
**Fitness index (*z*–score)** ***	-0.55 (-1.69–1.33)

*denotes using median (25^th^-75^th^ percentile range).

**denotes using percentages.

***sum of all physical fitness test *z*-scores.

Spearman coefficient showed strong correlations between left (*r* = 0.70; *p*<0.001) and right (*r* = 0.70; *p*<0.001) foot and the risk of falls. Partial coefficients remained significant after adjusting for age, body-mass index, foot pain and fitness index for both left (*r* = 0.61; *p*<0.001) and right (*r* = 0.58; *p*<0.001) foot.

Table 2 shows the associations between the left foot rotation and the risk of falls. In unadjusted model, higher foot rotation was associated with higher risk of falls (*β* = 0.14, *p*<0.001). Among other covariates, more frequent foot pain and lower fitness index were associated with higher risk of falls. In model adjusted for age, body-mass index, foot pain and fitness index, higher foot rotation remained significantly associated with higher risk of falls (*β* = 0.10, *p*<0.001).

**Table 2:** The associations between foot rotation (left foot) and the risk of falls in older women (*N* = 120).

Study variables	Model 1*			Model 2**		
	β***	95% CI	*p—*value	β***	95% CI	*p—*value
**Foot rotation**	**0.14**	**0.12–0.17**	**<0.001**	0.10****	**0.08–0.12**	**<0.001**
**Age**	0.01	-0.03–0.04	0.871	-0.01	-0.03–0.02	0.621
**Body—mass index**	-0.01	-0.06–0.04	0.621	0.01	-0.03–0.03	0.825
**Foot pain**	**0.59**	**0.15–1.04**	**0.009**	**0.28**	**0.00–0.57**	**0.049**
**Fitness index**	**-0.30**	**-0.36–0.24**	**<0.001**	**-0.17**	**-0.23–0.11**	**<0.001**

*unadjusted model (each predictor was put separately into the model).

**adjusted for age, body–mass index, foot pain and fitness index.

***unstandardized *β* coefficient.

**** The *β* coefficient of 0.10 means that for every 1 degree increase in foot rotation, the falls risk score increases by 0.10.

Table 3 shows the associations between the right foot rotation and the risk of falls. In unadjusted model, higher foot rotation was associated with higher risk of falls (*β* = 0.14, *p*<0.001). Among other covariates, more frequent foot pain and lower fitness index were associated with higher risk of falls. In model adjusted for age, body-mass index, foot pain and fitness index, higher foot rotation remained significantly associated with higher risk of falls (*β* = 0.10, *p*<0.001). Foot pain was no longer significantly associated with risk of falls in the fully adjusted model.

**Table 3:** The associations between foot rotation (right foot) and the risk of falls in older women (*N* = 120).

Study variables	Model 1*			Model 2**		
	β***	95% CI	*p—*value	β***	95% CI	*p—*value
**Foot rotation**	**0.14**	**0.12–0.17**	**<0.001**	0.10****	**0.07–0.13**	**<0.001**
**Age**	0.01	-0.03–0.04	0.871	-0.01	-0.03–0.02	0.575
**Body—mass index**	-0.01	-0.06–0.04	0.621	0.02	-0.02–0.05	0.287
**Foot pain**	**0.59**	**0.15–1.04**	**0.009**	0.15	-0.14–0.45	0.309
**Fitness index**	**-0.30**	**-0.36–0.24**	**<0.001**	**-0.19**	**-0.25–0.14**	**<0.001**

*unadjusted model (each predictor was put separately into the model)

**adjusted for age, body–mass index, foot pain and fitness index.

***unstandardized *β* coefficient

**** The *β* coefficient of 0.10 means that for every 1 degree increase in foot rotation, the falls risk score increases by 0.10.

## Discussion

The main purpose of the study was to determine the associations between foot rotation for both feet and the risk of falls in community-dwelling older adults. Our study shows a few important findings: (1) higher foot rotation in both feet is associated with higher risk of falls and (2) when we adjust for age, body-mass index, foot pain and fitness index, higher foot rotation in both feet remains associated with higher risk of falls.

Our results of increased risk of falls in those participants with deviated foot functions are in line with previous studies [9–13]. Specifically, a study by Mecagni et al. [13] showed that lower ankle range of motion was significantly associated with lower score of Performance-Oriented Mobility Assessment and functional reach scores. Another two studies have shown that a reduced dorsiflexion may increase the risk of falls by impairing balance and the ability to perform functional tests [11, 12]. A prospective-cohort study by Menz et al. [10] has shown that people who previously fell exhibit decreased ankle flexibility, more severe hallux valgus deformity, decreased plantar tactile sensitivity, and decreased toe plantar flexor strength. Similar findings have been found in adults with established rheumatoid arthritis, where fallers report greater self-reported foot impairment [9]. Of note, we observed both a non-significant association between foot pain in right foot and the risk of falls in the fully adjusted model (Table 3). Since our sample size was relatively small and was based on a healthy population of older adults without reported foot problems, we speculate that the aforementioned shortcomings might have underpowered the association. Also, in both unadjusted and adjusted model, fitness index was the strongest predictor of falls, mitigating the association between foot pain and the risk of falls. Finally, we additionally collected the data regarding preferred leg and found that all of 120 participants reported having right foot as ‘dominant’. We speculate, that ‘non-dominant’ leg is not as active as dominant one, leading to greater balance asymmetry and representing a contributing factor for falls among older individuals, which has been previously documented [18].

As mentioned in the ‘Introduction’ section, foot is the only body part that contacts the ground [7], making it the most essential structure and function. Foot structure and function in older adults are often characterized by flatter feet, intrinsic foot muscle weakness, altered plantar pressure loading patterns during walking, and reduced plantar tactile sensitivity [19]. Such deviations may lead to pain and discomfort, which unable older people to walk normally and increase the risk of health-related consequences, including higher incidence of falls. One modifiable factor that has been associated in lowering falls risk and reducing heath care costs is exercise [20]. Thus, the condition of having extreme foot rotation should be of important interest for special strategies and policies detecting such individuals and recruiting them to physical activity and performance programs.

This study has several limitations. First, by using a cross-sectional design, we cannot conclude the causality of the association. Second, we based our findings on a relatively small sample of participants (*N* = 120), and larger sample size may provide with somewhat different strength of the association. Third, we based our study on a sample living in the urban part of the country, speaking Croatian and only White race. Fourth, we studied relatively healthy older women and the associations between the level of foot rotation and the risk of falls might be differently relevant for less healthy older adults. Fifth, our study only included women and by including men, associations might have been different, and we could make the generalizability for both sexes. Finally, we did not test for foot structure (arch height) or additional functions (muscle activation or tactile sensitivity), which might have also brought different associations. Future prospective studies with larger sample size and detailed collection of foot structure and function variables need to be perform, in order to establish causal associations.

## Conclusions

Our study shows that higher foot rotation is associated with higher risk of falls in older women.

Although it was significant, the association between foot rotation and falls risk was very small and possibly not clinically important.

## Supporting information

S1 Rawdata (XLSX)
